# Investigating the association between stressful life events and the risk of myocardial infarction and mortality: the Tromsø study

**DOI:** 10.1186/s12872-025-05269-4

**Published:** 2025-12-24

**Authors:** Geir Fagerjord Lorem, Kjersti Lillevoll, Maja-Lisa Løchen, Tom Wilsgaard, Jens C. Thimm, Catharina Elisabeth Arfwedson Wang, Henrik Schirmer

**Affiliations:** 1 Department of Psychology, UiT The Arctic University of Norway, Tromsø, 9037 Norway; 2 Department of Clinical Medicine, UiT The Arctic University of Norway, Tromsø, Norway; 3 Department of Community Medicine, UiT The Arctic University of Norway, Tromsø, Norway; 4 Centre for Crisis Psychology, University of Bergen, Bergen, Norway; 5 Department of Cardiology, University Hospital of North Norway, Tromsø, Norway; 6 Department of Cardiology, Akershus University Hospital, Lørenskog, Norway; 7 Institute of Clinical Medicine, Campus Ahus, University of Oslo, Oslo, Norway

**Keywords:** Stressful life events, Myocardial infarction, Cardiovascular disease, Mortality, Epidemiology, Psychosocial stress, Risk factors, Cohort study, Mental distress, Tromsø study

## Abstract

**Background:**

Psychosocial stress is increasingly recognized as an independent risk factor for cardiovascular disease. However, the long-term impact of cumulative stressful life events on the risk of myocardial infarction (MI) and mortality in the general population remains underexplored. This study investigates the association between lifetime exposure to stressful life events and the risk of non-fatal MI, MI-related mortality, and all-cause mortality.

**Methods:**

We analyzed data from 21,830 participants aged ≥ 40 in the Tromsø Study (1994–2020). Exposure to stressful life events was assessed retrospectively in 2015–2016 and categorized into groups based on the total number of reported events. Cox proportional hazards models were used to estimate hazard ratios for non-fatal MI, MI-related mortality, and all-cause mortality, adjusting for age, sex, lifestyle, treatment, and biological factors.

**Results:**

Participants reporting four or more stressful life events experienced non-fatal MI on average 3.7 years earlier, MI-related mortality 12.3 years earlier, and all-cause mortality 9.5 years earlier than those without exposure to stressful life events. Higher exposure to stressful life events was associated with increased risk of non-fatal MI (hazard ratio 1.06 per additional event; 95% confidence interval: 1.01–1.10), MI-related mortality (hazard ratio 1.25; 95% confidence interval: 1.07–1.46), and all-cause mortality (hazard ratio 1.14; 95% confidence interval: 1.08–1.20). These associations persisted after adjustment for traditional cardiovascular risk factors.

**Conclusions:**

Lifetime exposure to stressful life events is associated with risks of MI, MI-related mortality, and all-cause mortality. Our findings highlight the importance of considering psychosocial stress in cardiovascular risk assessments and support the integration of screening for stressful life events into preventive cardiology.

**Supplementary Information:**

The online version contains supplementary material available at 10.1186/s12872-025-05269-4.

## Highlights

• Lifetime exposure to stressful life events is associated with risks of non-fatal myocardial infarction (MI), MI-related mortality, and all-cause mortality.

• Individuals with four or more stressful life events experienced MI on average 3.7 years earlier and had significantly higher mortality risk.

• The associations remained significant after adjustment for traditional cardiovascular risk factors, underscoring the independent impact of psychosocial stress.

• The findings support incorporating psychosocial stress assessment into cardiovascular risk management.

**Supplementary Information:**

The online version contains supplementary material available at 10.1186/s12872-025-05269-4.

## Background

The assessment and prevention of myocardial infarction (MI) and cardiovascular disease (CVD) focus primarily on modifiable physiological and lifestyle risk factors such as blood pressure, cholesterol levels, cigarette smoking, diabetes, and obesity [1]. Psychological factors such as exposure to stressful life events (SLEs) may cause mental distress that is directly linked to MI both via direct biological effects and indirect impacts through lifestyle risk factors such as smoking and poor adherence [1–3]. Despite declining incidence, MI remains a major public health concern, occurring even without modifiable risk factors [4]. A growing body of literature points to psychosocial stress as a CVD risk factor [5–8]. An extensive, prospective cohort study with two measurement points found a moderate but significant increase in incident MI and all-cause mortality risk in individuals with high stress levels with no prior infarction after adjusting for modifiable CVD risk factors. In that study, a composite measure of psychological stress related to potentially stressful life events and chronic stress was used [9]. Findings from studies on stress-related mental disorders, such as posttraumatic stress disorder (PTSD), suggest a bidirectional relationship between the disorders and CVD, where stress-related disorders may both increase the risk of developing CVD and emerge as a common psychiatric consequence of CVD events [10]. A population-based study with a 27-year follow-up found that individuals diagnosed with a stress-related disorder had a significantly elevated risk of CVD, with a hazard ratio of 1.71 in the first year following diagnosis and 1.36 thereafter. The study also provided more substantial evidence for an increased risk of early-onset MI in individuals under 50. The investigation did not account for modifiable risk factors such as smoking, hypertension, obesity, or physical inactivity [11]. A meta-analysis of studies on PTSD and CVD that did account for these risk factors yielded comparable results that suggest a cumulative effect of SLEs. In a study on health outcomes among cardiac patients, repeated lifetime trauma exposure significantly increased the risk of adverse outcomes [12, 13]. Studies of adverse childhood events indicate that the sequelae of such events extend far into adulthood [14–16].

Our study used 30 years of longitudinal panel data from the Tromsø Study database [17], the Norwegian National Cause of Death Registry, and adjudicated MI events from the University Hospital of North Norway discharge diagnosis registry. Utilizing a population-based cohort from Tromsø makes our findings generalizable to the general population, unlike studies that typically focus on high-risk groups or specific patient populations or have limited follow-up. The present study extends beyond cardiovascular outcomes, such as MI, to provide a more comprehensive perspective on the impact of SLEs on physical and mental health and associated risk factors and behaviors.

We aimed to investigate the cumulative impact of SLEs by examining lifetime exposure to SLEs and their association with MI outcomes and mortality. Our primary hypothesis centered on the notion that exposure to SLEs represented a risk factor for the early onset of MI and acted as an independent mortality risk factor post-MI. Additionally, we aimed to explore how SLEs influenced general health, mental well-being, cardiovascular risk factors, and health-related behaviors. Our secondary hypothesis was that CVD risk factors and health-related behaviors mediate the association between SLEs and MI events.

## Methods

The Tromsø Study is an extensive, ongoing population-based cohort study initiated in 1974 in the municipality of Tromsø in Northern Norway [18]. Originally launched to address the region’s high mortality rate from CVD, the study has expanded into a comprehensive investigation of chronic diseases and health conditions over five decades. The Tromsø Study has conducted seven waves, each gathering data on lifestyle factors and biomarkers. Mental health data collection began with Tromsø4 in 1994-95, significantly expanding the scope of the study. In this wave, 26,878 participants were enrolled, with a response rate of 71.8%. Subsequent waves, i.e., Tromsø5 (2001), Tromsø6 (2007-08), and Tromsø7 (2015-16), continued this trajectory with varying numbers of respondents and response rates. Tromsø7 included new participants as well as individuals from previous cohorts. It included 21,069 respondents aged 40 years and above, with a response rate of 65.0% [19]. We excluded participants with missing values on stressful life events (*n* = 239; 1.1% of the total sample), leaving 20,830 participants for the primary analyses. For additional information about the Tromsø Study, please visit https://uit.no/research/tromsostudy. The baseline for our study was the first available data point from Tromsø4–7, depending on when the participant entered the study. The assessment of the SLEs in Tromsø7 was thus a retrospective self-reported trauma assessment rather than a true baseline. Participants were asked about lifetime trauma exposure, but the temporal relationship between SLEs and MI events remains unclear.

### Measurements

Time and cause of death data were sourced from the Norwegian National Cause of Death Registry, known for its near-complete coverage [20]. The registration extended from baseline until the end of the follow-up period in 2020. The first non-fatal MI was identified by linking to the discharge diagnosis registry of the University Hospital of North Norway, including inpatient and outpatient cases. An independent endpoint committee conducted the adjudication of both in-hospital and out-of-hospital events. They used medical records, medical notes, autopsy records, and death certificates for their evaluations. A unique 11-digit national identification number facilitated linkage to national and local diagnosis registries. Cases of coronary heart disease were identified by referring to the discharge diagnosis registry of the University Hospital of North Norway, the sole hospital in the area. This involved searching for the codes I20 to I25, I46, R96, R98, and R99 of the International Classification of Diseases, 10th Revision (ICD-10). The committee used the criteria of the Modified World Health Organization Monitoring Trends and Determinants in Cardiovascular Disease (MONICA) and the MONICA Risk, Genetics, Archiving, and Monograph (MORGAM) studies. These criteria included clinical symptoms and signs, electrocardiogram findings, levels of cardiac biomarkers, and autopsy reports when applicable. The biomarkers considered were creatine kinase, its myocardial fraction (creatine kinase-MB), and troponin T, the latter since 1999. It is important to note that increases in biomarkers associated with revascularization procedures were not classified as MIs [17, 21].

Participants were asked to answer the following question about eleven SLEs: *“Have you ever experienced one of the following events?“*. Response options were: *‘No*,*’ ‘Yes*,* before age 18*,*’ ‘Yes*,* after age 18*,*’* and *‘Yes*,* in the past year.’* The only exception was *childhood neglect*, which had a binary response of *‘Yes’* or *‘No.’* Lifetime exposure was calculated as the total of all reported SLEs. A complete list of SLEs and descriptive statistics can be found in Supplementary Table 1. The questionnaire has previously been published [22] and all questions are included in the supplementary file. It is worth noting that we lack precise information on whether SLEs reported as occurring after age 18 occurred before or after the participant’s first MI event. To minimize reversed causation, since an MI itself is likely to be perceived as a traumatic illness, the analyses distinguish between pre-MI and post-MI reports of SLEs, excluding life-threatening illness as an SLE, when analyzing MI risk.

Our analyses included sex and age as confounding variables. Treatment, biological factors such as systolic blood pressure, cholesterol levels, diabetes (defined as HbA1c >6.5%), and health-related behaviors (smoking and physical activity) were considered as possible mediating factors. At the research site, specially trained personnel collected information about the physical and biological characteristics of the participants. Weight and height were measured separately and subsequently used to calculate BMI in kg/m². Non-fasting blood samples sent to the laboratory provided total cholesterol and HbA1c data. The personnel also measured blood pressure, including systolic blood pressure. A dichotomous variable in Tromsø4 measured daily smoking, but “previously smoked” was added to Tromsø5–7. Data on self-reported hard physical activity, where participants started to sweat and became out of breath, were collected through the questionnaire, which asked participants to report their weekly average of physical activity during leisure time over the past year. The responses were categorized as inactive (0 times a week), light (less than once a week), moderate (once or twice a week), and intensive (three or more times a week) physical activity. The physical activity variable was further recoded into inactive (0) and active (≥ once a week on average). Self-rated health, reflecting the participant’s overall health situation, was evaluated on a scale ranging from “very poor” (1) to “very good” (5).

The Hopkins Symptom Checklist-10 (HSCL-10) is a brief self-report instrument designed to assess mental distress, particularly symptoms of anxiety and depression [23]. The recommended cut-off level of 1.85 was used to identify persons with significant symptom levels, and the subthreshold was defined as ≥ 1.40, < 1.85 [24].

### Statistical analysis

Our study’s conceptual framework postulates that SLEs are associated with non-fatal MI events, MI mortality, and all-cause mortality. In our analysis, the primary outcomes of interest are the occurrence of the first-ever MI event, MI-specific mortality, and all-cause mortality, with SLEs designated as the independent variable of interest. We conducted all statistical analyses using STATA (version 18; StataCorp LLC, College Station, TX, USA).

Descriptive characteristics: Descriptive statistics, including means, standard deviations, and percentages, were calculated for variables stratified by groups. Participants were categorized into groups based on SLE exposure: no SLE, one, two, three, and four or more SLEs. This categorization yielded SLE groups of roughly equal size. Analyzing participant profiles based on SLE exposure helps to highlight disparities in health outcomes and risk factors, emphasizing the potential impact of experiencing SLEs. We examined baseline characteristics across SLE groups using chi-square tests for tabulated data and ANOVA for continuous variables.

Health and risk factor trajectories: In this stage, we aimed to delineate health and risk factor trajectories leading up to and following an MI event. We used a retrospective time series approach, where data from Tromsø4–7 were organized into repeated measures. The time variable was defined as the difference between the attendance date and the date of the first MI event. We employed a locally weighted regression (LOWESS) to model the mean variation over time. This non-linear technique allowed us to create smooth curves from irregular data points, circumventing issues arising from inappropriate linearity assumptions.

The Cox proportional hazards model with non-fatal MI as the outcome was used to examine the association between exposure to SLEs and the time to the first non-fatal MI in four models. SLEs were added as a linear variable. Model 1 included age and sex. Model 2 included Model 1 covariates + lifestyle factors (daily smoking, physical exercise). Model 3 included Model 2 covariates + treatment factors (blood pressure-lowering and lipid-lowering drugs). Model 4 included Model 3 covariates + biological factors (blood pressure, cholesterol, body mass, long-term blood sugar). Participants who had experienced a non-fatal MI were considered cases, while those who had remained event-free were treated as non-cases. The baseline for all participants was their first attendance date at Tromsø4–6, and time was measured as age in days. Participants were followed until the date of MI or participation in Tromsø7, whichever came first. Prevalent cases at baseline were excluded from the analysis. The SLE variable was adjusted in the main models to exclude life-threatening illness as a stressful life event to avoid MI being classified as both an exposure and an outcome. To control for the effect of retrospectively asking about illness trauma, we also show the calculations for illness trauma and total SLE (i.e., including illness trauma). Hazard ratios with 95% confidence intervals (CIs) were reported, indicating the risk of experiencing a non-fatal MI. The proportional hazards assumption was evaluated using Schoenfeld residuals, and no violations were detected.

The Cox proportional hazards model with all-cause and MI mortality as outcomes was used to examine the association between exposure to SLEs and time to all-cause and MI mortality. The event of interest for all-cause mortality was defined as death from any cause after the Tromsø7 study (2015–2016), while MI mortality was defined as death explicitly attributed to MI. The baseline for all participants was their attendance date at Tromsø7, with time measured as age in days. Participants were followed up until death, or the end of the study on December 31, 2020, whichever came first.

## Results

### Sample characteristics

Table 1 presents participant characteristics in Tromsø7, categorized by SLE exposure. The data showed that mental distress and self-rated health worsened as the number of SLEs increased. Generally, women reported more mental health symptoms and SLEs, while men showed fewer favorable biomedical risk factors. Individuals with four or more SLEs had higher rates of obesity and smoking, while blood pressure, cholesterol, and long-term blood sugar showed minor variations. After the MI, individuals with SLE exposure demonstrated the most significant improvements in health-related behaviors, including smoking cessation. In total, 20,830 participants were included in the primary analyses, after excluding 239 individuals with missing SLE data from the 21,069 Tromsø7 participants. Among these participants, 5251 (48.7% women) reported no SLEs, 4916 (51.2% women) reported one SLE, 4144 (54.6% women) reported two SLEs, 2721 (54.9% women) reported three SLEs, and 3798 (55.6% women) reported four or more SLEs. Women reported higher exposure to emotional and interpersonal SLEs, including sexual abuse and childhood neglect. Men reported higher exposure to physical or violent SLEs, such as life-threatening illnesses or accidents, direct exposure to violence, and frightening or dangerous experiences (cf. Supplementary Table 2). Notably, individuals with four or more SLEs exhibited an earlier onset of MI (mean difference = 3.7 person-years), earlier MI-related mortality (mean difference = 12.3 person-years), and earlier all-cause mortality (mean difference = 9.5 person-years), as compared with those without SLE exposure (cf. Supplementary Table 3).

**Table 1:** Sample characteristics by stressful life events (SLEs). The Tromsø study 2015/2016

	No SLE *N =* 5251			One SLE *N =* 4916			Two SLEs *N =* 4144			Three SLEs *N =* 2721			≥Four SLEs *N =* 3798		*P*-value
Age (Mean/SD)	58.4	(11.7)	57.8		(11.5)	57.6		(11.3)	57.3		(11.4)	55.1		(10.7)	< 0.001
Sex (Count/%)															
Female	2558	48.7%	2519		51.2%	2263		54.6%	1494		54.9%	2110		55.6%	< 0.001
Male	2693	51.3%	2397		48.8%	1881		45.4%	1227		45.1%	1688		44.4%	
Self-rated health (Count/%)															
Poor	177	3.4%	186		3.8%	208		5.1%	150		5.6%	409		10.9%	< 0.001
Not so good	1238	23.8%	1106		22.7%	1036		25.2%	758		28.1%	1213		32.2%	
Good	2884	55.4%	2794		57.3%	2258		54.9%	1427		53.0%	1770		47.0%	
Very good	909	17.5%	792		16.2%	611		14.9%	359		13.3%	371		9.9%	
Mental health symptoms (Count/%)															
No symptoms	2344	46.9%	1839		37.9%	1205		29.3%	683		25.3%	656		17.4%	< 0.001
Some symptoms	1771	35.4%	1870		38.5%	1630		39.7%	1048		38.8%	1236		32.8%	
Sub-threshold symptoms	686	13.7%	891		18.4%	948		23.1%	679		25.2%	1115		29.6%	
Significant symptoms	195	3.9%	253		5.2%	325		7.9%	288		10.7%	760		20.2%	
Hypertension (Count/%)															
No	3076	58.6%	2887		58.7%	2485		60.0%	1594		58.6%	2443		64.3%	< 0.001
Yes	2175	41.4%	2029		41.3%	1659		40.0%	1127		41.4%	1355		35.7%	
Hyperlipidemia (Count/%)															
No	2153	41.0%	1924		39.1%	1587		38.3%	1042		38.3%	1357		35.7%	< 0.001
Yes	3098	59.0%	2992		60.9%	2557		61.7%	1679		61.7%	2441		64.3%	
Body mass index (Count/%)															
< 18.5 kg/m2	27	0.5%	30		0.6%	27		0.7%	9		0.3%	22		0.6%	< 0.001
18.5–24.9 kg/m2	1770	33.8%	1571		32.0%	1369		33.1%	862		31.8%	1085		28.7%	
25–29.9 kg/m2	2316	44.3%	2169		44.2%	1783		43.1%	1207		44.5%	1591		42.0%	< 0.001
> 30 kg/m2	1119	21.4%	1134		23.1%	957		23.1%	635		23.4%	1086		28.7%	
Diabetes (Count/%)															
No	4949	94.2%	4615		93.9%	3928		94.8%	2568		94.4%	3566		93.9%	0.121
Yes	302	5.8%	301		6.1%	216		5.2%	153		5.6%	232		6.1%	
Daily smoking (Count/%)															
Current smoker	631	12.1%	578		11.9%	555		13.5%	395		14.7%	702		18.7%	< 0.001
Previous smoker	2178	41.8%	2136		43.8%	1855		45.2%	1213		45.0%	1774		47.2%	
Never smoked	2398	46.1%	2163		44.4%	1697		41.3%	1085		40.3%	1284		34.1%	
Physical activity (Count/%)															
Inactive	707	13.5%	637		13.0%	566		13.7%	384		14.1%	615		16.2%	< 0.001
Active	4544	86.5%	4279		87.0%	3578		86.3%	2337		85.9%	3183		83.8%	

### MI event risk and mortality risk

We observe that individuals with higher SLE exposure were more likely to experience MI at a younger age. The first MI event was observed at 28.3 years of age, with the last event recorded at 100.4 years of age. Over the total follow-up period of 1,282,554.5 person-years, 901 MI events were recorded. Regarding mortality, the first recorded death occurred at age 42.5, while the last death was reported at age 101.3. During the entire follow-up period of 1,322,650.3 person-years, there were 533 recorded deaths, resulting in a mortality rate of 0.025 deaths per person-year. Our analyses did not reveal any significant interaction between SLE and sex in the models.

The results from the Cox proportional hazards models in the first line of Table 2 show the relationship between SLEs and the risk of non-fatal MI. We found a modest but significant association between SLE exposure and MI across all the models. Since an MI event may be experienced as a life-threatening illness, it was not included as an SLE in the primary model. In Model 1 (adjusted for age and sex), the hazard ratio (HR) was 1.06 per unit increment in the number of SLEs (95% CI: 1.02–1.10) and this association remained consistent across the fully adjusted Model 4 (HR 1.06, 95% CI: 1.02–1.10), which also accounted for lifestyle, treatment, and biological factors. However, when the analysis included life-threatening illness as an SLE, the association between SLE exposure and MI became notably stronger, as shown in Supplementary Table 4.

**Table 2:** Adjusted hazard ratios for incident non-fatal myocardial infarction, all-cause mortality, and MI-related mortality associated with the number of stressful life events in the Tromsø study

	Model 1		Model 2		Model 3		Model 4	
	HR	CI	HR	CI	HR	CI	HR	CI
Non-fatal MI	1.06	[1.02,1.10]	1.05	[1.01,1.10]	1.07	[1.02,1.11]	1.06	[1.02,1.10]
All-cause mortality	1.15	[1.09,1.21]	1.15	[1.10,1.22]	1.15	[1.09,1.21]	1.14	[1.08,1.20]
MI-related mortality	1.33	[1.16,1.53]	1.31	[1.13,1.51]	1.29	[1.12,1.50]	1.25	[1.07,1.46]

Primary analyses exclude illness-related trauma; sensitivity analyses including these events are presented in Supplementary Table 4. 95% confidence intervals in brackets. The hazard rate corresponds to each additional unit increase in the number of SLEs

Model 1 included age and sex

Model 2 included model 1 covariates + lifestyle factors (daily smoking, physical exercise)

Model 3 included model 2 covariates + treatment factors (BP-lowering and lipid-lowering drugs)

Model 4 included model 3 covariates + biological factors (BP, cholesterol, body mass, long-term blood sugar)

*MI* Myocardial infarction, *HR* Hazard ratio, *CI* Confidence interval, *BP* Blood pressure, *SLE* Stressful life events

A similar pattern was observed for all-cause mortality. Participants with no SLEs had a mean age at death of 78.5 years (SD = 10.4), while those with four or more SLEs had a notably younger mean age at death (69.0 years, SD = 11.6). For MI-related mortality, participants with no SLEs had a mean age of 80.9 years (SD = 10.5) at the time of MI-related death, while those with four or more SLEs were significantly younger, with an average age of 68.6 years (SD = 12.0). Table 2 also presents the results from the proportional hazard models, with the primary outcome being all-cause mortality. Our analysis indicated that experiencing SLEs throughout one’s life was associated with a hazard ratio of 1.15 (95% CI, 1.09–1.21). This pattern persisted across all models.

Table 2 also displays the results obtained from the proportional hazard models for MI-related deaths. Our analysis indicated that experiencing multiple SLEs throughout one’s life was linked to an increased risk of MI mortality. The hazard ratio was 1.33 for each additional SLE (95% CI: 1.16–1.53). However, there was some attenuation when controlling for CVD risk and treatment factors (HR 1.25, 95% CI: 1.07–1.46).

It was a common observation across all models that lifestyle, treatment, and biological factors did not radically alter the adverse effects of SLEs. Additionally, we found that the hazard ratio for illnesses related to retrospectively reported trauma was more pronounced than that for full exposure to SLEs, even when illness-related trauma was included.

### Health and risk trajectories before and after MI

**Fig. 1:**
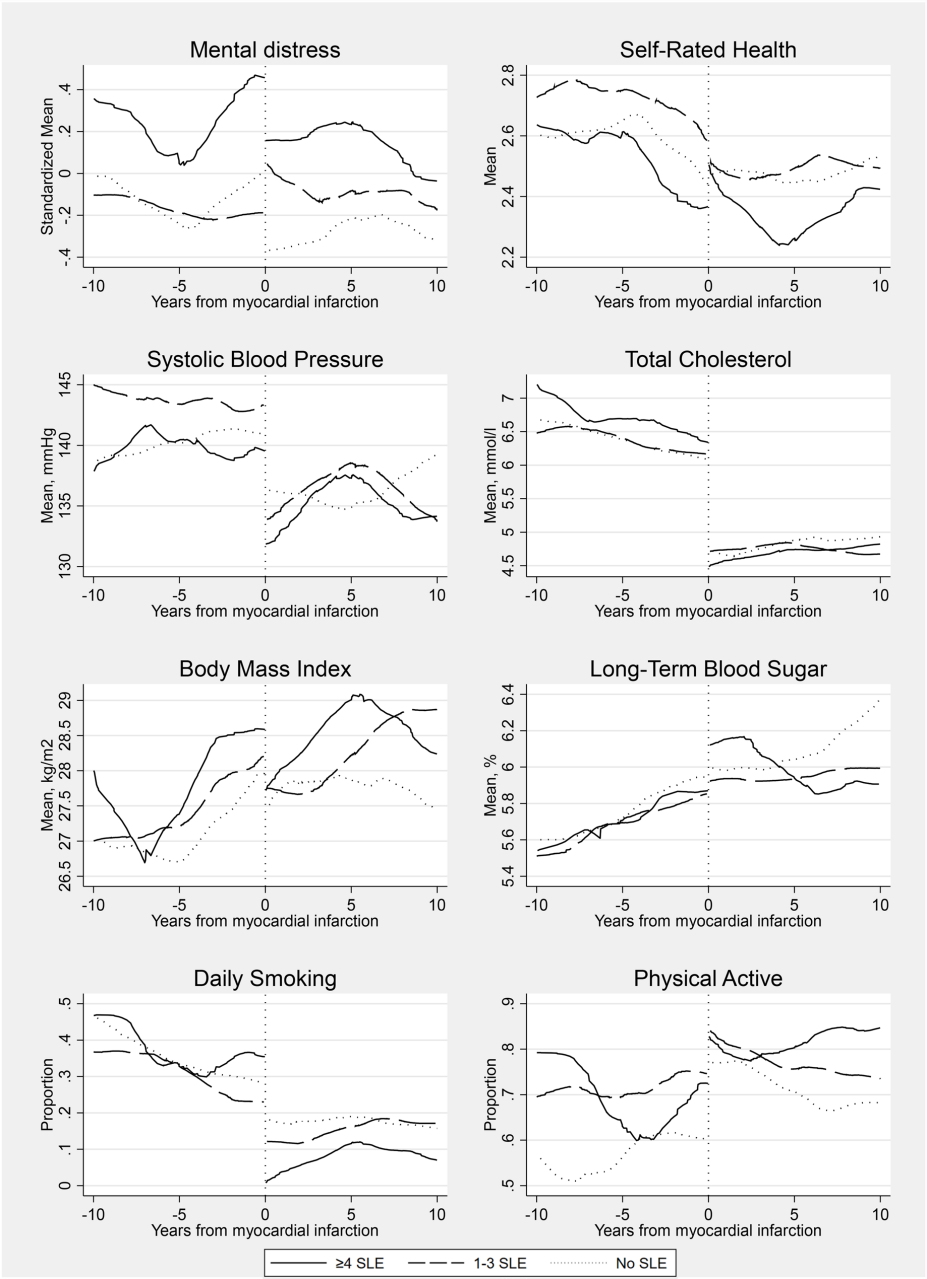
Locally Weighted Scatterplot Smoothing (LOWESS) showing health and risk factor trajectories in the years before and after myocardial infarction by lifetime numbers of stressful life events. We included individuals with a confirmed diagnosis of MI (*N* = 901). Time was relative to the date of the first MI. The Y-axis shows the mean variance among the groups over the different time points. Separate models were fitted before and after the first MI event. Mental distress = HSCL-10 standardized; Long-term blood sugar = HbA1c is measured as a percentage of glycated hemoglobin; Physical activity = Active vs. inactive (proportion); Daily smoking = Current vs. non-smoker (proportion); BMI is measured in kg/m^2^; Blood pressure is measured in mmHg, Total cholesterol is measured in mmol/l

We found noticeable differences in various health and modifiable risk factors among individuals with SLE exposure before experiencing a heart attack (MI). Figure 1 shows the changes in these factors over the ten years preceding and following the heart attack. There appeared to be a shift in most outcomes around five years before the first heart attack, particularly in self-reported health, blood pressure, cholesterol, and smoking. However, body mass and long-term blood sugar levels remained on less favorable trajectories. SLE exposure was linked to increased mental distress (*p* < 0.001) and daily smoking (*p* < 0.01). After the heart attack, SLE exposure was still associated with mental distress (*p* < 0.001), daily smoking (*p* < 0.05), and physical activity (*p* < 0.01). Our analyses did not reveal any significant interaction between SLE and time in the models. This suggests a consistent impact of SLE exposure across pre- and post-heart attack periods without substantial temporal variation.

## Discussion

Our findings consistently demonstrate that the adverse effects of SLEs on cardiovascular outcomes persist even after adjusting for lifestyle factors, medical treatment, and biological risk factors. This robustness across all models underscores the association of SLE exposure in shaping long-term health risks, particularly of MI and all-cause mortality. The fact that adjustments for known cardiovascular risk factors did not attenuate the effect of SLEs suggests that mechanisms beyond traditional pathways, such as stress-related physiological dysregulation, may contribute to the observed associations.

It is known that SLEs may trigger the activation of the hypothalamus-pituitary-adrenal (HPA) axis, leading to the release of stress hormones in response to repeated or prolonged stress exposure [25]. Stress responses that are adaptive in the short term ultimately become detrimental to health in the long run. A significant body of research suggests that repeated stress activation may lead to dysregulation of the HPA axis, manifesting as hyperactivation. Hyperactivation, associated with increased cortisol activity, has been linked to hypertension [26], coronary artery calcification [27], and even cellular aging [28]. Hypoactivation, associated with blunted cortisol response, has received less research attention, but there is some evidence that it can be linked to obesity and symptoms of anxiety and depression [29]. However, the exaggerated cardiovascular and cortisol responsiveness to stress has been robustly linked to unfavorable cardiovascular outcomes. SLEs are also associated with heightened sympathetic activity and reduced parasympathetic regulation, leading to higher resting heart rate, increased blood pressure variability, and decreased heart rate variability [30, 31]. These autonomic disturbances predict hypertension, arrhythmias, and cardiovascular mortality [32], further compounding cardiovascular risk [33].

A recent review examined the potential role of sleep variables (i.e., short sleep duration, sleep disturbance, and sleep deprivation) as mediators of CVD risk factors. The authors argued that the evidence suggested a possible mechanism whereby low sleep quality, short sleep duration, and sleep disruptions might increase CVD risk through sympathetic hyperarousal, hypertension, proinflammatory responses, and endothelial dysfunction. Clinical interventions addressing sleep disturbance as a CVD risk factor are a promising line of research that remains to be pursued [34].

Third, adverse health behaviors associated with SLE exposure, such as smoking, consumption of unhealthy food, overeating, and sedentary lifestyle, can exacerbate biological risk factors such as elevated cholesterol, high blood pressure, obesity, and diabetes [35]. We observed that the SLE group demonstrated increasing BMI, daily smoking, and diabetes. Blood pressure and cholesterol levels started high but improved leading up to the MI event. After the event, these factors were, on average, within recommended limits, probably due to secondary prevention risk management. Nevertheless, the association between prior SLEs and health outcomes remained significant even when considering traditional CVD risk factors, such as high blood pressure, cholesterol levels, and unhealthy behaviors.

We found that the hazard ratio for retrospectively reported trauma-related illnesses was even more pronounced than for overall exposure to SLE, regardless of whether trauma-related diseases were included in the total SLE measure. This suggests that individuals who have experienced severe illnesses, especially those perceived as life-threatening, may be more vulnerable to adverse outcomes. However, the retrospective nature of SLE reporting raises concerns about reverse causation, where individuals who have already experienced a serious illness, such as an MI, might be more likely to classify it as a traumatic event retrospectively. As a result, the perceived impact of SLEs on cardiovascular outcomes may be exaggerated when life-threatening illnesses are included in the analysis.

Other studies have indicated that individuals who had experienced four or more SLEs were likely to meet the criteria for PTSD [10, 36]. This could be a critical factor in considering their risk of CVD. Trauma treatment can help to alleviate the long-term effects of stress and enhance overall well-being. This raises the question of whether trauma therapy should be incorporated as part of a comprehensive strategy for preventing CVD in high-risk groups. Psychosocial interventions can also target health behaviors associated with SLEs and CVD risk, such as smoking cessation, promoting physical activity, and encouraging healthy eating habits. One study showed that achievable and realistic improvements in health metrics could prevent nearly 15% of MIs [37]. Motivational interviewing and behavior change techniques can help individuals adopt and maintain positive lifestyle changes [30, 31, 38]. The patterns in our study indicate both positive and negative developments in the years leading up to an MI event. Specifically, individuals with multiple SLEs exhibited beneficial health behaviors, such as increased physical activity and a decline in levels of traditional risk factors, including improved blood pressure and cholesterol levels, approximately three to four years before the MI event. However, this positive trajectory coexists with concerns about developments in self-reported health, mental distress, BMI, and smoking.

Women reported a higher overall exposure to SLE, especially emotional and interpersonal traumas. However, men displayed less favorable biomedical profiles, characterized by higher rates of obesity and smoking. This difference may result from several interrelated factors: (1) Reporting differences (e.g., women might be more inclined to disclose certain SLEs). (2) Biological differences (e.g., there may be variations in how stress responses are managed biologically). (3) Gender-specific coping strategies (e.g., men might adopt less healthy behaviors such as smoking, poor diet, as coping mechanisms in response to stress, while women may express distress more through psychological symptoms). Despite these different pathways, higher exposure to SLEs was linked to worse health outcomes for both genders, including earlier onset of MI and increased mental distress. Nonetheless, in both sexes, higher SLE exposure was linked to earlier MI and greater mental distress, indicating multiple pathways to elevated cardiovascular risk. These patterns warrant further research to clarify the underlying mechanisms.

While current clinical guidelines acknowledge psychosocial factors as potential risk modifiers, it is paradoxical that they do not provide a clear framework for treatment or include SLE exposure as a risk factor [1]. Although much research remains to be conducted to fully understand the mechanisms involved, the evidence of experiences of SLEs seems compelling. It would seem pertinent to include SLE screening in CVD risk assessments and consider the need for clinical interventions.

### Strengths and limitations of this study

The study used cohort data from over 21,000 participants in the Tromsø Study, providing robust and extensive longitudinal data to assess the long-term effects of SLE exposure on cardiovascular outcomes. The analysis controlled for a wide range of CVD risk factors, including age, sex, cholesterol levels, blood pressure, smoking status, and diabetes, thereby strengthening the validity of the findings by reducing the impact of potential confounders. However, it is essential to acknowledge the limitations of our study.

First, the SLEs were reported retrospectively, which may have introduced both survival and recall bias, potentially impacting the accuracy of the data. Self-reporting is prone to memory bias when compared to registry-based data, as it can be influenced by various factors, including psychological distress [32] and individual personality traits [39]. Participants who did not survive long enough for the Tromsø7 study may have differed from those included, as SLE and MI were associated with higher mortality. This could have led to an underestimation of the connection between SLEs and negative health outcomes.

Second, SLEs occurring after age 18 were measured without any additional information regarding the timing of those events. This lack of temporal context may have affected the precision and accuracy of the results, highlighting the need for careful interpretation. Although we have identified a specific effect of SLEs on MI outcomes, further research is required to establish a causal link, as the temporal sequence between SLEs and MI remains unclear. Additionally, we need more information about mediating factors that may influence this relationship, such as socioeconomic status, lifestyle choices, and pre-existing health conditions.

Third, we excluded illness trauma from the SLE measure in the main analysis in Table 2, but we conducted a sensitivity analysis to address reversed causation by also including illness trauma. The results are presented in Supplementary Table 4. This approach helped clarify whether this specific category disproportionately influences the association between trauma exposure and cardiovascular outcomes. The persistence of significant effects, even when illness trauma was included, suggested that other forms of trauma also contribute significantly to increased cardiovascular risk. Supplementary Table 5 presents the hazard ratios for SLEs occurring before the age of 18. We observe a similar trend to that seen with lifetime exposure; however, trauma from illness does not appear to be an independent factor. Furthermore, while stressful childhood events are associated with all-cause and MI-related deaths, their relationship with the occurrence of the first myocardial infarction is not statistically significant.

We partially addressed this by focusing on non-fatal MI cases and using population-based recruitment to reduce selection bias (see the supplementary material for the sensitivity analysis). Despite this, our ability to establish the exact timing of stress exposure and cardiovascular events remains limited. Self-reported measures are essential in epidemiological research, capturing subjective experiences often missed in other records. When appropriately applied and adjusted, they can provide valuable insights at the population level. However, the retrospective assessment of SLEs in Tromsø7 remains a limitation, as we cannot establish the exact timing of SLE exposure in relation to the onset of MI. Future studies that use prospective trauma assessments are necessary to confirm these findings and better understand the directionality of the observed associations.

The shared detrimental patterns in self-reported health, mental distress, BMI, and smoking across the SLE groups could potentially signify a common external triggering event that advances the trajectory toward an MI event. Similarly, the beneficial patterns in physical activity, smoking, and especially blood pressure and total cholesterol, may imply that these factors have been addressed and mitigated to reduce CVD risk. While such improvements could be related to behavioral changes following SLE exposure, another plausible explanation is that they reflect indirect effects of increased contact with doctors and other health services, leading to earlier detection and medical management of elevated blood pressure and cholesterol. Although our data do not record the timing of SLEs relative to the cardiovascular event, we could hypothesize that SLE exposure in adulthood might be a tipping point toward an MI event in high-risk individuals with a history of repeated SLE exposures. Alternatively, it could indicate that their resilience may have been exhausted due to previous repeated SLE encounters.

## Conclusion

Our findings indicate that repeated exposure to SLEs is associated with risks of non-fatal MI, MI-related mortality, and all-cause mortality. Individuals with multiple SLE exposures experienced MI and related mortality at a younger age, and this association persisted even after adjusting for traditional CVD risk factors, such as hypertension and diabetes. Interestingly, while SLE exposure was linked to adverse health outcomes, it also coincided with positive behavioral changes such as increased physical activity and smoking cessation, indicating that trauma may encourage individuals to engage more with healthcare. However, these changes did not mitigate the long-term cardiovascular risks associated with SLEs. The adverse health effects of SLEs were observed in both genders, highlighting the broad impact of psychosocial stress on CVD risk.

### Clinical and public health implications

In view of these strong associations, we recommend integrating SLE exposure screening into CVD risk assessments. Current guidelines recognize psychosocial stressors as risk modifiers but lack a structured approach to addressing mental health history, which is a critical gap in cardiovascular prevention. In addition to traditional risk management, psychosocial interventions for trauma-exposed individuals could help lower long-term cardiovascular risks. Future research should explore the effectiveness of targeted interventions for these individuals.

## Supplementary Information

Supplementary Material 1.

## Data Availability

The dataset supporting the findings in this article is available upon application to the Tromsø Study. Please follow the steps presented on their online page: https://uit.no/research/tromsostudy.

## Abbreviations

BMI: Body mass index
CI: Confidence interval
CVD: Cardiovascular disease
HSCL-10: Hopkins Symptoms Checklist–10
MI: Myocardial infarction
OR: Odds ratio
SLE: Stressful life events
