# Supplementary material for: Investigating the association between stressful life events and the risk of myocardial infarction and mortality: the Tromsø study
Source: BMC Cardiovasc Disord. 2025 Dec 24;25:874. doi: 10.1186/s12872-025-05269-4 (PMC12729713; doi:10.1186/s12872-025-05269-4)
Supplement: Supplementary file 1 — Supplementary Material 1. [file 12872_2025_5269_MOESM1_ESM.docx]

Supplementary Table 1. Stressful life events questions with distribution.

| Question | Have not experienced | Yes, before age 18 | Yes, after age 18 | Yes, last year | HR First MI | HR All-cause death | HR MI- related death |
| --- | --- | --- | --- | --- | --- | --- | --- |
| Have you ever experienced one of the following events? A life-threatening illness or a severe accident (for example, fire, work accident, or car accident) | 15,910 76.4% | 1,095 5.3% | 3,650 17.5% | 389 1.9% | 1.75 [1.54,1.99] | 1.63 [1.37,1.94] | 1.97 [1.14,3.41] |
| Have you ever experienced one of the following events? Been exposed to violence (for example, hit, kicked, beaten, robbed, or threatened with a firearm) | 17,629 84.6% | 1,213 5.8% | 2,121 10.2% | 93 0.45% | 1.00 [0.82,1.23] | 2.23 [1.75,2.83] | 2.96 [1.57,5.60] |
| Have you ever experienced one of the following events? Been exposed to sexual abuse, i.e., sexual actions against your will | 18,846 90.5% | 1,500 7.2% | 592 2.8% | 8 0.04% | 0.80 [0.57,1.12] | 1.33 [0.92,1.93] | 2.33 [0.96,5.70] |
| Have you ever experienced one of the following events? Been called negative things, marginalized, threatened, or bullied by schoolmates, fellow students, or coworkers over a more extended period | 16,422 78.8% | 3,314 15.9% | 1,167 5.6% | 295 1.4% | 0.94 [0.78,1.13] | 1.79 [1.44,2.23] | 2.07 [1.11,3.86] |
| Witnessed someone close to you being exposed to violence or sexual abuse (for example, hit, kicked, beaten, robbed, or threatened with a firearm) | 18,909 90.8% | 901 4.3% | 1,039 5.0% | 107 0.51% | 0.99 [0.75,1.30] | 1.79 [1.31,2.44] | 1.67 [0.62,4.46] |
| Experienced something else that was frightening, dangerous, or violent (for example, natural disaster, war, terror attack, held captive) | 19,117 91.8% | 612 2.9% | 1,058 5.18% | 91 0.44% | 0.98 [0.79,1.20] | 0.82 [0.64,1.04] | 0.80 [0.38,1.70] |
| Death of a close one and difficulty accepting the loss, yearning for the deceased, and intense emotional pain related to the loss. | 14,012 67.3% | 1,064 5.1% | 5,295 25.4% | 842 4.0% | 1.30 [1.14,1.47] | 1.23 [1.04,1.44] | 1.62 [1.00,2.64] |
| Received painful medical treatment due to sickness or severe injury when in hospital. | 18,596 89.3% | 713 3.4% | 1,409 6.8% | 178 0.9% | 1.31 [1.07,1.60] | 1.50 [1.16,1.95] | 1.30 [0.56,3.01] |
| Received painful medical treatment from the dentist. | 15,880 76.2% | 3,833 18.4% | 1,200 5.8% | 147 0.71% | 1.14 [0.99,1.33] | 1.37 [1.12,1.66] | 1.67 [0.96,2.92] |
| That someone close to you had a life-threatening illness or was exposed to a severe accident. | 13,337 64.0% | 889 4.3% | 5,977 28.7% | 1,129 5.4% | 0.96 [0.84,1.11] | 1.14 [0.95,1.37] | 1.89 [1.16,3.10] |
| Failure of care in childhood, i.e. not having received the necessary food, clothing, protection, and care/love from parents/caregivers | 19,204 93.1% | 1,415 6.9% | n/a | n/a | 1.03 [0.76,1.40] | 1.40 [0.98,2.00] | 2.56 [1.10,5.99] |

Note: The proportion is within each question category. Since a person may report several stressful life events within each question, the sum is not necessarily 100%. We used Cox proportional hazards regression to examine the association between exposure to SLEs and the time to first non-fatal MI. The baseline for all participants was their first attendance date at Tromsø4–6, and time was measured as age in days. Participants were followed until the date of MI or date of participation in Tromsø7, whichever came first. We also used Cox proportional hazards regression to examine the association between exposure to SLEs and the time to all-cause and MI mortality. The event of interest for all-cause mortality was defined as death from any cause after Tromsø7 (2015–2016), while MI mortality was defined as death attributed explicitly to MI. The baseline for all participants was their attendance date at Tromsø7. Participants were followed up until the date of death or the end of the study on December 31, 2020, whichever came first.

Supplementary Table 2. Prevalence of stressful life events stratified by sex

| Stressful life event | Group | Mean | SD | [95% conf. | interval] | p-value |
| --- | --- | --- | --- | --- | --- | --- |
| Life-threatening illness or accident | |  |  |  |  |  |
| Female | | 0.208 | 0.421 | 0.200 | 0.216 | <0.001 |
| Male | | 0.279 | 0.471 | 0.270 | 0.288 |  |
| Exposed to violence | |  |  |  |  |  |
| Female | | 0.134 | 0.358 | 0.127 | 0.141 | <0.001 |
| Male | | 0.193 | 0.429 | 0.184 | 0.201 |  |
| Sexual abuse | |  |  |  |  |  |
| Female | | 0.158 | 0.391 | 0.151 | 0.165 | <0.001 |
| Male | | 0.035 | 0.186 | 0.031 | 0.038 |  |
| Been called negative things, marginalized, threatened, or bullied | |  |  |  |  |  |
| Female | | 0.207 | 0.434 | 0.199 | 0.215 | 0.1208 |
| Male | | 0.216 | 0.449 | 0.207 | 0.225 |  |
| Witnessed someone close to you being exposed to violence or sexual abuse | |  |  |  |  |  |
| Female | | 0.099 | 0.314 | 0.093 | 0.104 | 0.0012 |
| Male | | 0.085 | 0.300 | 0.079 | 0.091 |  |
| Experienced something else that was frightening, dangerous, or violent | |  |  |  |  |  |
| Female | | 0.074 | 0.268 | 0.069 | 0.079 | <0.001 |
| Male | | 0.093 | 0.298 | 0.087 | 0.099 |  |
| Death of a close one and difficulty accepting the loss | |  |  |  |  |  |
| Female | | 0.386 | 0.519 | 0.377 | 0.396 | <0.001 |
| Male | | 0.283 | 0.474 | 0.273 | 0.292 |  |
| Received painful medical treatment | |  |  |  |  |  |
| Female | | 0.116 | 0.328 | 0.109 | 0.122 | 0.001 |
| Male | | 0.101 | 0.307 | 0.095 | 0.107 |  |
| Received painful dental treatment | |  |  |  |  |  |
| Female | | 0.244 | 0.452 | 0.236 | 0.253 | 0.843 |
| Male | | 0.246 | 0.454 | 0.237 | 0.255 |  |
| Someone close had a life-threatening illness or severe accident. | |  |  |  |  |  |
| Female | | 0.411 | 0.525 | 0.401 | 0.420 | <0.001 |
| Male | | 0.330 | 0.503 | 0.320 | 0.339 |  |
| Failure of care during childhood | |  |  |  |  |  |
| Female | | 0.082 | 0.274 | 0.077 | 0.087 | <0.001 |
| Male | | 0.054 | 0.226 | 0.049 | 0.058 |  |

Note: We performed t tests on the equality of means comparing men and women where the mean represents the prevalence of each stressful life event question.

Supplementary Table 3. Age at first MI, at all-cause death, and at MI-related death by stressful life events. The Tromsø Study 2015/2016

|  | No SLE  *N=*5251 | | 1 SLE *N=*4916 | | 2 SLEs *N=*4144 | | 3 SLEs *N=*2721 | | ≥4 SLEs *N=*4037 | |
| --- | --- | --- | --- | --- | --- | --- | --- | --- | --- | --- |
| Age at first MI | 58.6 | (11.6) | 58.0 | (11.3) | 57.7 | (11.1) | 57.4 | (11.1) | 54.9 | (10.0) |
| Age at MI death | 80.9 | (10.5) | 77.0 | (10.2) | 77.5 | (10.8) | 76.3 | (10.2) | 68.6 | (12.0) |
| Age at all-cause death | 78.5 | (10.4) | 78.7 | (10.1) | 75.0 | (11.3) | 75.1 | (11.2) | 69.0 | (11.6) |

Supplementary Table 4. Adjusted hazard ratios for incident non-fatal myocardial infarction, all-cause death, and MI-related death associated with stressful life events, The Tromsø Study

| **First non-fatal MI** | **Model 1** | | **Model 2** | | **Model 3** | | **Model 4** | |
| --- | --- | --- | --- | --- | --- | --- | --- | --- |
|  | HR | CI | HR | CI | HR | CI | HR | CI |
| **SLEs without illness trauma** | 1.06 | [1.02,1.10] | 1.05 | [1.01,1.10] | 1.07 | [1.02,1.11] | 1.06 | [1.02,1.10] |
| **Illness trauma** | 1.75 | [1.54,1.99] | 1.75 | [1.54,1.99] | 1.76 | [1.55,2.00] | 1.72 | [1.51,1.96] |
| **SLEs including illness trauma** | 1.10 | [1.06,1.14] | 1.09 | [1.05,1.13] | 1.11 | [1.07,1.15] | 1.10 | [1.06,1.14] |
| **All-cause death** | **Model 1** | | **Model 2** | | **Model 3** | | **Model 4** | |
|  | HR | CI | HR | CI | HR | CI | HR | CI |
| **SLEs without illness trauma** | 1.15 | [1.09,1.21] | 1.15 | [1.10,1.22] | 1.15 | [1.09,1.21] | 1.14 | [1.08,1.20] |
| **Illness trauma** | 1.63 | [1.37,1.94] | 1.65 | [1.39,1.97] | 1.60 | [1.34,1.91] | 1.58 | [1.32,1.88] |
| **SLEs including illness trauma** | 1.10 | [1.06,1.14] | 1.09 | [1.05,1.13] | 1.11 | [1.07,1.15] | 1.10 | [1.06,1.14] |
| **MI-related death** | **Model 1** | | **Model 2** | | **Model 3** | | **Model 4** | |
|  | HR | CI | HR | CI | HR | CI | HR | CI |
| **SLEs without illness trauma** | 1.33 | [1.16,1.53] | 1.31 | [1.13,1.51] | 1.29 | [1.12,1.50] | 1.25 | [1.07,1.46] |
| **Illness trauma** | 1.97 | [1.14,3.41] | 1.96 | [1.12,3.42] | 1.89 | [1.07,3.31] | 1.78 | [1.00,3.17] |
| **SLEs including illness trauma** | 1.33 | [1.16,1.51] | 1.30 | [1.14,1.49] | 1.29 | [1.12,1.48] | 1.24 | [1.07,1.44] |

95% confidence intervals in brackets; MI = myocardial infarction; HR = hazard ratio; CI = confidence interval; BP = blood pressure; SLE=Stressful life events; SD=Standard deviation. Model 1 included age and sex. Model 2 included Model 1 covariates + lifestyle factors (daily smoking, physical exercise). Model 3 included Model 2 covariates + treatment factors (BP-lowering and lipid-lowering drugs). Model 4 included Model 3 covariates + biological factors (BP, cholesterol, body mass, long-term blood sugar).

Supplementary Table 5. Adjusted hazard ratios for incident non-fatal myocardial infarction, all-cause death, and MI-related death associated with stressful life events occurring before the age of 18, The Tromsø Study

| **First non-fatal MI** | **Model 1** | | **Model 2** | | **Model 3** | | **Model 4** | |
| --- | --- | --- | --- | --- | --- | --- | --- | --- |
|  |  |  |  |  |  |  |  |  |
| **SLEs without illness trauma** | 1.06 | [0.99,1.14] | 1.06 | [0.98,1.14] | 1.09 | [1.01,1.18] | 1.09 | [1.01,1.17] |
| **Illness trauma** | 0.77 | [0.52,1.13] | 0.76 | [0.52,1.12] | 0.79 | [0.53,1.15] | 0.81 | [0.55,1.19] |
| **SLEs including illness trauma** | 0.77 | [0.52,1.13] | 0.76 | [0.52,1.12] | 0.79 | [0.53,1.15] | 0.81 | [0.55,1.19] |
| **All-cause death** | **Model 1** | | **Model 2** | | **Model 3** | | **Model 4** | |
|  |  |  |  |  |  |  |  |  |
| **SLEs without illness trauma** | 1.21 | [1.11,1.33] | 1.24 | [1.13,1.36] | 1.24 | [1.13,1.35] | 1.23 | [1.12,1.34] |
| **Illness trauma** | 1.38 | [0.91,2.07] | 1.32 | [0.87,1.99] | 1.33 | [0.88,2.01] | 1.32 | [0.87,2.00] |
| **SLEs including illness trauma** | 1.21 | [1.11,1.31] | 1.22 | [1.13,1.33] | 1.22 | [1.12,1.33] | 1.21 | [1.12,1.32] |
| **MI-related death** | **Model 1** | | **Model 2** | | **Model 3** | | **Model 4** | |
|  |  |  |  |  |  |  |  |  |
| **SLEs without illness trauma** | 1.45 | [1.14,1.83] | 1.40 | [1.08,1.80] | 1.39 | [1.07,1.79] | 1.34 | [1.02,1.76] |
| **Illness trauma** | 2.46 | [0.88,6.88] | 2.57 | [0.91,7.23] | 2.53 | [0.90,7.12] | 2.60 | [0.92,7.37] |
| **SLEs including illness trauma** | 1.44 | [1.16,1.79] | 1.40 | [1.11,1.77] | 1.39 | [1.10,1.76] | 1.36 | [1.06,1.74] |

95% confidence intervals in brackets; MI = myocardial infarction; HR = hazard ratio; CI = confidence interval; BP = blood pressure; SLE=Stressful life events; SD=Standard deviation. Model 1 included age and sex. Model 2 included Model 1 covariates + lifestyle factors (daily smoking, physical exercise). Model 3 included Model 2 covariates + treatment factors (BP-lowering and lipid-lowering drugs). Model 4 included Model 3 covariates + biological factors (BP, cholesterol, body mass, long-term blood sugar).

In examining childhood experiences, we observe a similar pattern to that of lifetime exposure; however, illness trauma does not appear to be an independent factor. Additionally, the relationship between stressful childhood events and the occurrence of the first myocardial infarction (MI) is not significant, although it is associated with MI-related deaths.
